# Supplementary material for: Gastrointestinal Parasites of Dogs in Egypt: An Update on the Prevalence in Dakahlia Governorate and a Meta-Analysis for the Published Data from the Country
Source: Animals (Basel). 2023 Jan 31;13(3):496. doi: 10.3390/ani13030496 (PMC9913534; doi:10.3390/ani13030496)
Supplement: Supplementary file 1 [file animals-13-00496-s001.zip › animals-2073634-supplementary.pdf]

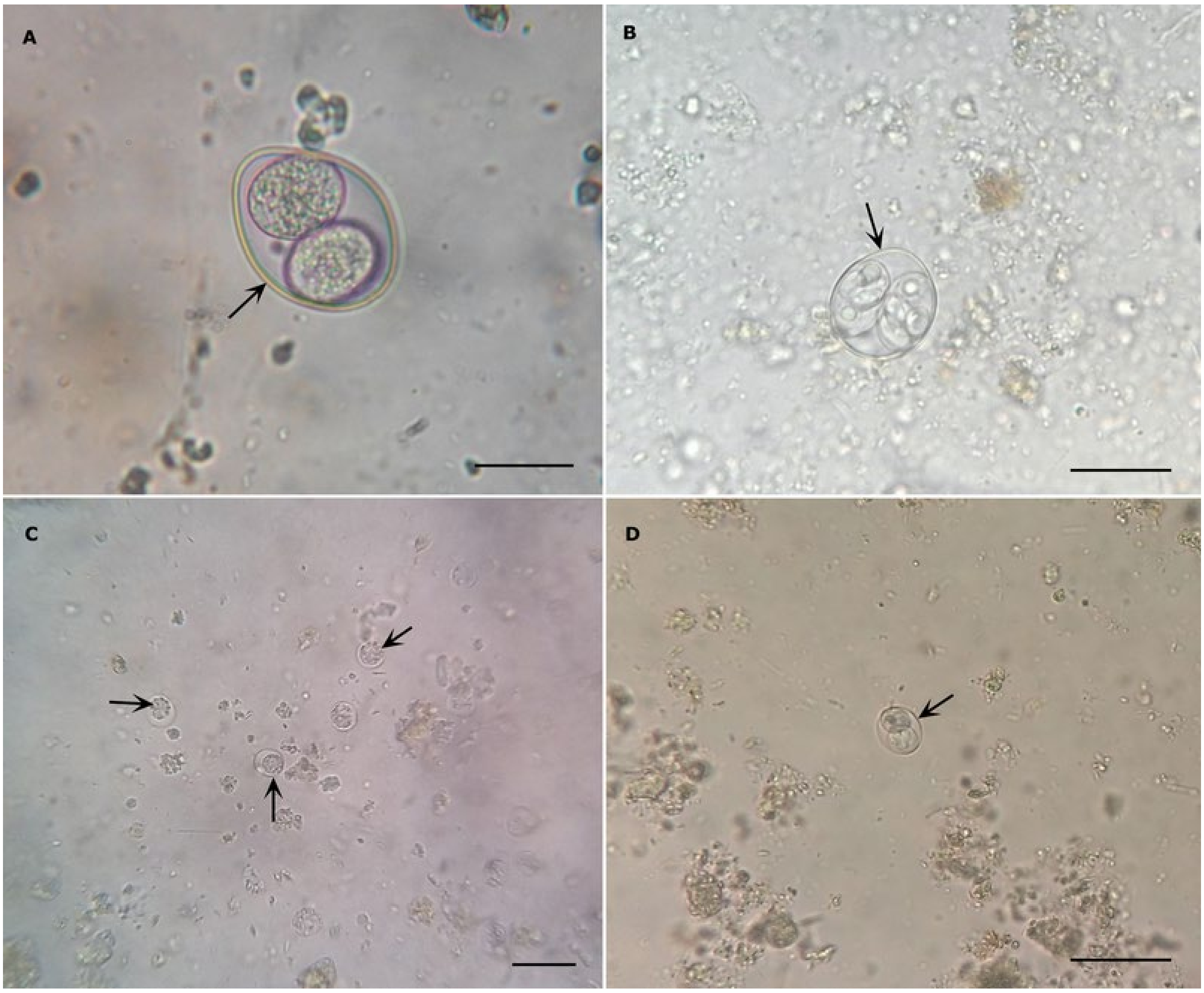

Figure S1. Photomicrographs for oocysts (arrow/s) of coccidian parasites detected in feces of stray dogs in Dakahlia governorate, Egypt. **A**. *Cystoisospora canis*. **B**. *Cysatoisospora ohioensis*. **C**. *Neospora caninum*-like unsporulated oocysts. **D**. *Neospora caninum*-like sporulated oocyst. Scale bar in all parts = 20 µm.

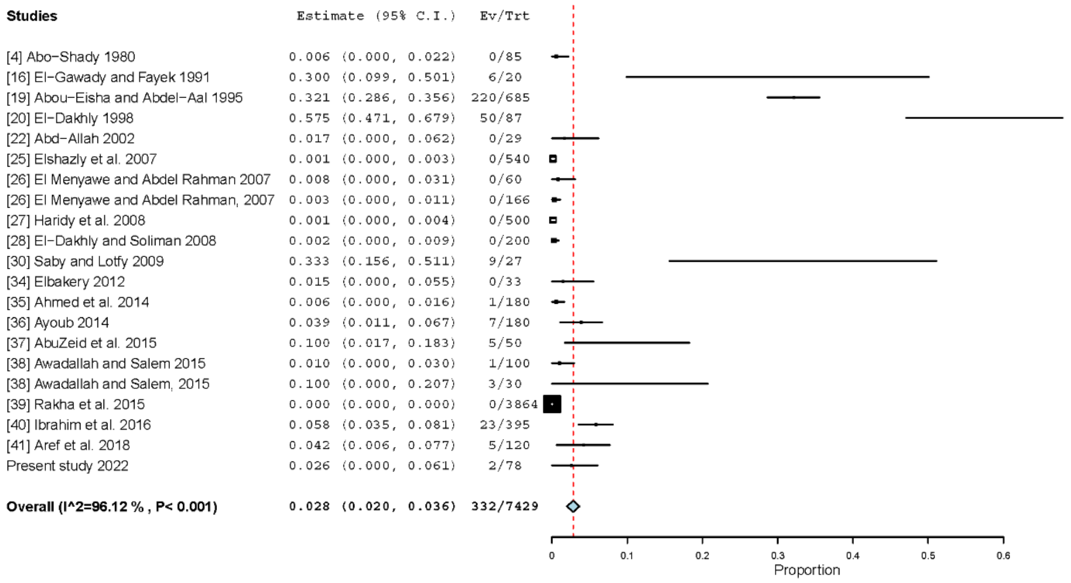

Figure S2. Forest plot diagram for random effects in the meta-analysis of the prevalence of *T. leonina* in dogs from Egypt. The length of line indicates 95% confidence interval of each study and the middle point of each line refers to the prevalence. Diamond refers to the overall prevalence.

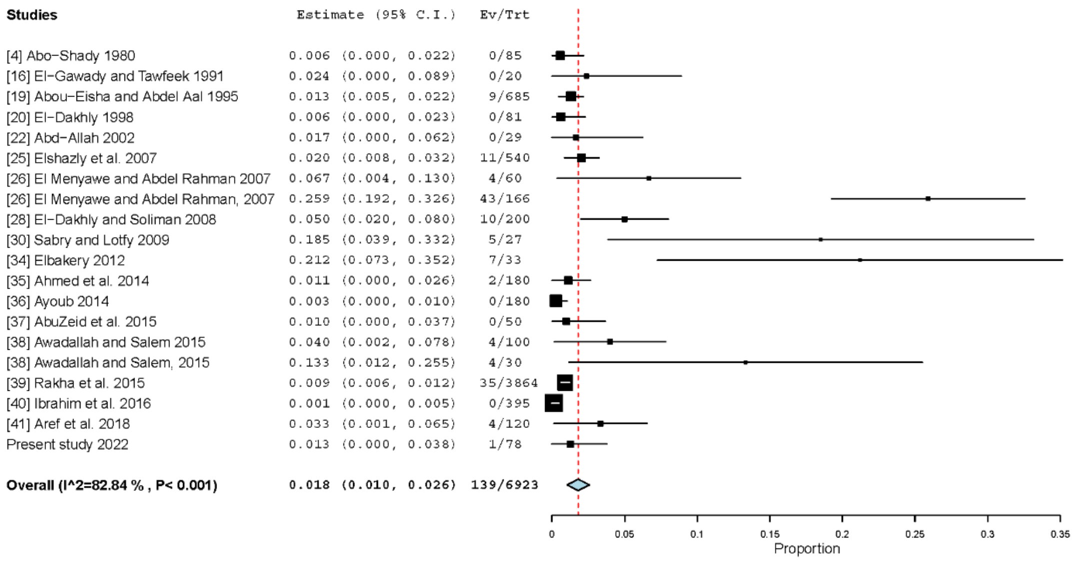

Figure S3. Forest plot diagram for random effects in the meta-analysis of the prevalence of hookworms in dogs from Egypt. The length of line indicates 95% confidence interval of each study and the middle point of each line refers to the prevalence. Diamond refers to the overall prevalence.

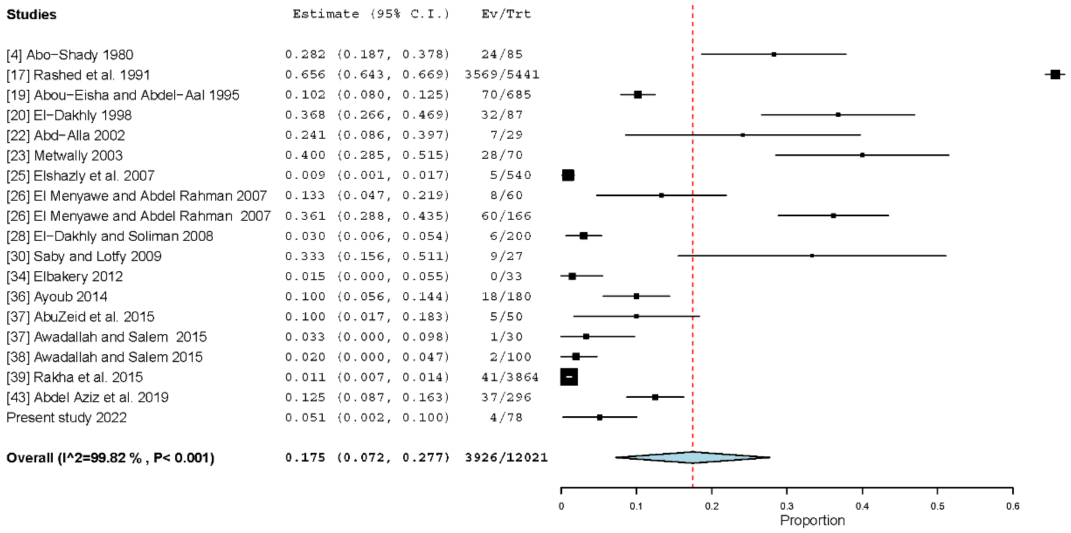

Figure S4. Forest plot diagram for random effects in the meta-analysis of the prevalence of *Taenia* spp. in dogs from Egypt. The length of line indicates 95% confidence interval of each study and the middle point of each line refers to the prevalence. Diamond refers to the overall prevalence.

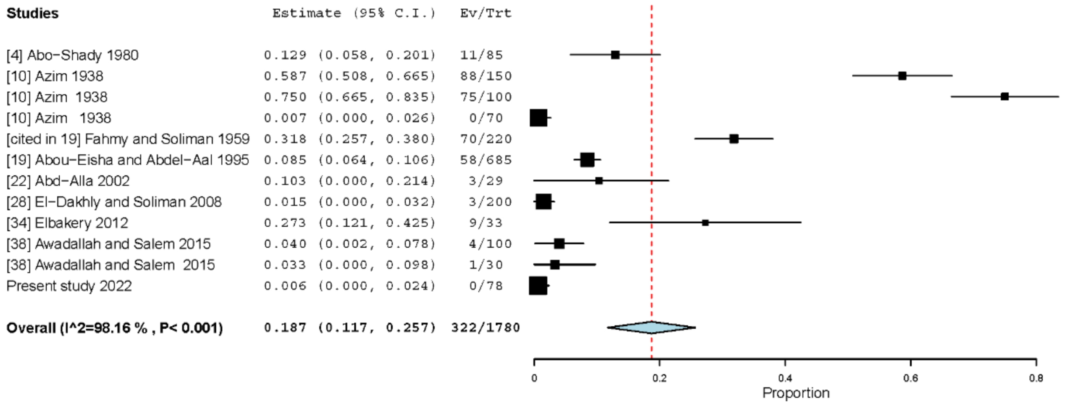

Figure S5. Forest plot diagram for random effects in the meta-analysis of the prevalence of *H. heterophyes* in dogs from Egypt. The length of line indicates 95% confidence interval of each study and the middle point of each line refers to the prevalence. Diamond refers to the overall prevalence.
